# Toward a Comparative Study of Collective Memory and Citizenship Among Older Adults With Dementia

**DOI:** 10.1093/geroni/igaa057.1069

**Published:** 2020-12-16

**Authors:** Jessica Robbins

**Affiliations:** Wayne State University, Detroit, Michigan, United States

## Abstract

This poster explores new directions for understanding possibilities for citizenship among older adults with dementia, who often experience loss of citizenship and diminishment of personhood due to their diagnosis. Drawing on data from two distinct ethnographic studies—one on memory and personhood in a day center for people with Alzheimer’s disease in Poland, and the other on gardening and wellbeing among older African Americans without cognitive impairment in Detroit—this poster asks how the collective past may shape experiences of dementia and possibilities for citizenship in the present. In Poland, practices of remembering involving collective memory can sustain personhood and foster ties of relatedness among people with dementia. This apparent paradox between people with dementia’s loss of memory and their capacity to build social relations based on remembering can be resolved through expanding understandings of personhood to include practices of remembering involving collective pasts (e.g., shared national frameworks, embodied practices of sociality). In Detroit, gardening fosters connections with the past, as older African Americans are reminded of deceased loved ones through practices and the plants themselves. These intimate connections and everyday activities are situated in racialized histories of migration, disinvestment, and “revitalization,” even as they provide the means to cultivate life in the present. This poster concludes that the potential for collective pasts to generate life in the present can become evident through ethnographic research among people with dementia, and in particular, through studying gardening among people with dementia.

